# Single‐cell landscape of sex‐specific drivers of Alzheimer's disease

**DOI:** 10.1002/alz.71041

**Published:** 2025-12-28

**Authors:** Yiyang Wu, Kyle J. Travaglini, Mariano Gabitto, C. Dirk Keene, Amy R. Dunn, Catherine C. Kaczorowski, Philip L. De Jager, Vilas Menon, Julie A. Schneider, David A. Bennett, Logan Dumitrescu, Timothy J. Hohman

**Affiliations:** ^1^ Vanderbilt Memory and Alzheimer's Center Vanderbilt University Medical Center Nashville Tennessee USA; ^2^ Allen Institute for Brain Science Seattle Washington USA; ^3^ Department of Laboratory Medicine and Pathology University of Washington Seattle Washington USA; ^4^ The Jackson Laboratory Bar Harbor Maine USA; ^5^ Department of Neurology University of Michigan Ann Arbor Michigan USA; ^6^ Center for Translational and Computational Neuroimmunology Department of Neurology Columbia University Irving Medical Center New York New York USA; ^7^ Taub Institute for Research on Alzheimer's Disease and the Aging Brain Columbia University Irving Medical Center New York New York USA; ^8^ Rush Alzheimer's Disease Center Rush University Medical Center Chicago Illinois USA; ^9^ Vanderbilt Genetics Institute Vanderbilt University Medical Center Nashville Tennessee USA

**Keywords:** alzheimer's disease, dementia, RNA sequencing, sex difference, signaling pathway, single cell

## Abstract

**INTRODUCTION:**

We investigated sex‐specific gene expression associations with the neuropathology and cognitive manifestation of Alzheimer's disease (AD) leveraging single‐nucleus transcriptomic datasets including 2.84 million nuclei from the dorsolateral prefrontal cortex (DLPFC).

**METHODS:**

We delineated the full scope of sex‐specific transcript associations, differential gene expression, signaling pathway, and cell–cell communication network changes in eight major DLPFC cell types.

**RESULTS:**

Nine female‐specific associations were identified and replicated, involving *ADGRV1*, *OR3A3*, *IFI27L1*, *LYRM1*, *STAP2*, *TSTD2*, *PDYN*, and *TMEM50B*. We observed the preponderance of protective female‐specific associations in neurons. Sex‐specific genes were enriched in the immune‐, inflammation‐, and damage‐related stress‐response pathways. Six ITGB1‐mediated microglia‐specific incoming signals that may contribute to female‐specific risk of Aβ accumulation were also highlighted.

**DISCUSSION:**

Our study highlights the transcriptome‐wide, single‐cell landscape of sex‐specific molecular associations with AD neuropathology and cognitive decline, with identifying and replicating several female‐specific gene associations in neurons to help direct future mechanistic studies.

**Highlights:**

- Single‐nucleus transcriptomic association analysis identified 2660 sex‐specific associations involving 2110 genes with four AD endophenotypes.
- The majority of female‐specific associations link to better endophenotype outcomes were from neurons.
- Nine female‐specific associations were replicated, including *ADGRV1* and *OR3A3* with Aβ; *IFI27L1*, *LYRM1*, *STAP2*, and *TSTD2* with tau; *PDYN* with global cognition; and *TMEM50B* with longitudinal cognitive trajectory.
- Sex‐specific effect genes were enriched in the immune‐, inflammation‐, and damage‐related stress‐response pathways.
- Six ITGB1‐mediated microglia‐specific incoming signals may play roles in female‐specific risk for Aβ accumulation.

## 1 BACKGROUND

Alzheimer's disease (AD) has a higher prevalence rate in women, with women making up two‐thirds of AD cases.^1^, ^2^ While differences in lifespan contribute to the gender disparity in AD,^3^ multiple studies have found a higher age‐specific incidence rate of AD in females versus males, particularly at older ages,^4^, ^5^ although findings have been mixed.^6^, ^7^, ^8^ Women with AD tend to have more AD neuropathology,^9^, ^10^, ^11^ faster cognitive decline,^12^, ^13^ and faster brain atrophy^14^ and experience enhanced risk when carrying the APOE ε4 allele.^15^, ^16^ Despite carrying more pathology, multiple studies have shown that women have higher cognitive reserve and resilience compared to men, because it takes a higher burden of neocortical tau to clinically manifest disease.^17^, ^18^ In contrast, men with AD have a higher mortality rate and a greater comorbidity burden.^19^, ^20^, ^21^, ^22^, ^23^ Thus, there is a pressing need to better characterize the molecular factors that underlie such sex differences in the neuropathology and clinical manifestation of AD.

Our team has been particularly interested in identifying genetic factors that act in a sex‐specific manner to modify risk and resilience in AD. We and others have identified novel female‐specific and male‐specific drivers of AD neuropathology,^24^, ^25^ cognitive decline,^26^ and resilience to cognitive decline leveraging advanced genomic approaches.^27^ Additionally, we have provided a deep characterization of sex‐specific associations between APOE and AD neuropathology^28^ and cognitive decline.^16^ Together, our work has suggested that the sex‐specific genetic drivers of AD emerge largely downstream of Aβ, with a notable sex‐specific genetic architecture that contributes to tau deposition, neurodegeneration, and cognitive decline.^29^

In addition to the genomic evidence, multiple studies have characterized sex differences in AD using bulk multiomics measured in brain and blood tissue of humans and mice,^30^, ^31^, ^32^, ^33^ consistently highlighting that sex‐specific genes are enriched for immune response. The advancement of single‐cell/nucleus RNA sequencing (scRNA‐seq or snRNA‐seq) in recent years has facilitated a more complete dissection of the molecular mechanisms that contribute to sex differences in AD at single‐cell resolution. Although a few studies to date have investigated sex‐specific transcript associations with AD diagnosis at cellular resolution,^34^, ^35^, ^36^ no studies have provided robust replication of sex‐specific effects or investigated associations with AD endophenotypes that provide needed biological specificity.

We build on the current literature by leveraging a novel, large snRNA‐seq dataset (AD Knowledge Portal: syn2580853) derived from dorsolateral prefrontal cortex (DLPFC) tissue of 424 donors from the Religious Orders Study and the Rush Memory and Aging Project (ROS/MAP),^37^ and by performing an independent replication of sex‐specific associations utilizing an independent snRNA‐seq dataset from 84 donors in the Seattle Alzheimer's Disease Brain Cell Atlas (SEA‐AD) study.^38^ We move beyond sex dimorphic differential gene expression to identify sex‐specific transcript associations with four AD endophenotypes, including neuropathology and cognitive performance, while also providing robust statistical evidence of independent replication. Moreover, we also provide detailed sex differences in cell–cell communication alterations in the AD brain, along with biological pathways that show sex‐specific enrichment in the AD brain. The goal of this manuscript is to comprehensively characterize the transcriptome‐wide single‐cell landscape of sex‐specific molecular associations with AD neuropathological burden and longitudinal cognitive decline to reveal cell‐type‐specific drivers of the pathogenesis and progression of AD dementia.

## 2 METHODS

### 2.1 Study participants

The participants included in this study were from two longitudinal clinical‐pathological cohort studies including ROS/MAP. Each study was approved by the Institutional Review Board (IRB) of Rush University Medical Center. At enrollment, all participants were free of known dementia and agreed to annual clinical evaluation and brain donation.^39^, ^40^, ^41^

Independent replication was sought leveraging snRNA‐seq data from 84 donors in the SEA‐AD study.^42^ *Post mortem* brain tissue and donor metadata of SEA‐AD study were obtained via the University of Washington (UW) BioRepository and Integrated Neuropathology (BRaIN) laboratory from participants in the Kaiser Permanente Washington Health Research Institute Adult Changes in Thought (ACT) Study and the University of Washington Alzheimer's Disease Research Center (ADRC).

**RESEARCH IN CONTEXT**

1. **Systematic review**: We reviewed the literature using traditional sources (e.g., PUBMED and Google Scholar). Women have a twice higher prevalence of AD than men. Bulk omics analysis of brain tissues revealed sex‐specific molecular contributors, and the more recent single‐cell RNA sequencing technology made it possible to look into their cell‐type‐specific roles. However, none of these studies provided replications.
2. **Interpretation**: Our study highlighted nine female‐specific associations involving eight unique genes with AD neuropathologies and cognitive performance; the preponderance of protective female‐specific associations in neurons; the important roles of immune‐, inflammation‐, and damage‐related stress‐response pathways in contributing to sex differences; and six ITGB1‐mediated microglia‐specific incoming signals that may contribute to female‐specific risk of Aβ accumulation.
3. **Future directions**: Our study highlights the transcriptome‐wide, single‐cell landscape of sex‐specific molecular associations with AD endophenotypes. Further investigation will be needed to explore larger, genetically diverse cohorts and in more AD‐affected brain tissues.

### 2.2 Single‐nucleus RNA sequencing

**FIGURE 1:**
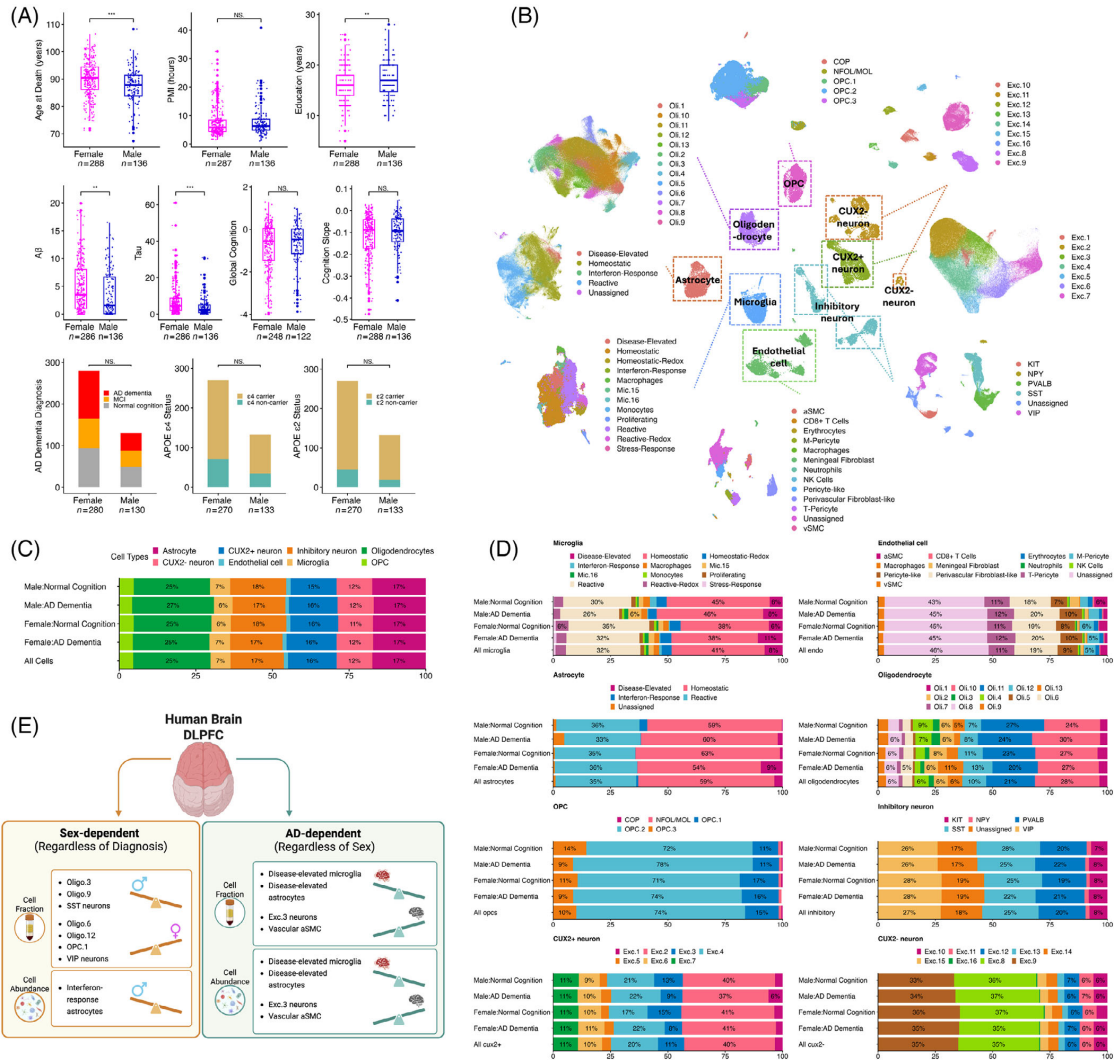
ROS/MAP single‐nucleus RNA sequencing (snRNA‐seq) cohort characteristics and cell composition comparison. (A) ROS/MAP snRNA‐seq cohort characteristics summary, showing age at death, *post mortem* interval (PMI), education length, Aβ, tau, global cognition at last visit, cognition slope, AD dementia diagnosis, and *APOE* ε2 and ε4 carrier status. ***p < 0.001, **p < 0.01, *p < 0.05. For *APOE* ε2 carrier, *APOE* ε4 carrier status, and diagnosis, statistical testing was done for the number of *APOE* ε2 carriers, *APOE* ε4 carriers, or AD dementia patients between sexes. (B) Joint UMAP, colored by major cell type. Composition of each major cell type was represented by subtype UMAP. (C) Proportion of each major cell type in ROS/MAP snRNA‐seq samples by sex and diagnostic groups. (D) The proportion of each subtype of each major cell type in ROS/MAP snRNA‐seq samples by sex and diagnostic groups. (E) Sex‐dependent or AD dementia diagnosis‐dependent cell composition changes. Panel (E) was created in BioRender. DLPFC, dorsolateral prefrontal cortex; NS, not significant. Aβ, amyloid beta; AD, Alzheimer's disease; APOE, apolipoprotein E; ROS/MAP, Religious Orders Study and the Rush Memory and Aging Project; snRNA‐seq, single‐nucleus RNA sequencing; UMAP, uniform manifold approximation and projection.

Single‐nucleus transcriptomes of DLPFC brain specimens derived from 465 ROS/MAP participants (AD Knowledge Portal Accession Number: syn31512863) were collected by the Rush ADRC and processed at Columbia University Medical Center, including sample processing, library preparation and sequencing of single nuclei, processing of snRNA‐seq reads, quality control, cell type classification, and cell subclass analysis. Detail methods can be found in a previous report.^43^ The post‐quality control (post‐QC) datasets contained 424 unique participants with a median of 3824 sequenced nuclei and 1.64 million nuclei in total. All cell type clusters were adapted from the original publication. Eight major cell types were clustered and used for all analyses, including astrocytes, vascular niches (referred to as “endothelial cells” in this article), microglia, oligodendrocytes, oligodendrocyte precursor cells (OPCs), inhibitory neurons, and CUX2+ and CUX2– excitatory neurons (Figure 1B). Excitatory neurons were split into two subgroups to increase computational efficiency (CUX2+ consisted of upper cortical layer 2–4 pyramidal neurons, and CUX2– consisted of all others deeper cortical layer pyramidal neurons). In addition, the original 67 cell type subgroups were used for the cell composition analyses. Genes with expression in at least 10% of all cells were used for downstream analyses. Cells were removed from downstream analyses if they had over 20,000 or less than 200 total RNA unique molecular identifiers (UMIs) or had over 5% mitochondrially mapped reads. RNA molecular count data were normalized and scaled by the sctransform R package^44^ (version 2) using “percent.mt” values for “vars.to.regress” parameter.

The snRNA‐seq dataset of SEA‐AD (syn52146347) consisted of 1.2 million nuclei derived from 84 donors’ *post mortem* DLPFC (Brodmann area 9) tissues. Cells were mapped to the National Institutes of Health's (NIH) Brain Research through Advancing Innovative Neurotechnologies (BRAIN) Initiative – Cell Census Network (BICCN)‐based cellular taxonomy,^45^ which resulted in 24 subclasses, including LAMP5 LHX6, LAMP5, PAX6, SNCG, VIP, SST CHODL, SST, PVALB, CHANDELIER, L2/3 IT, L6 IT, L4 IT, L5 IT, L5 ET, L6 CT, L6B, L6 IT *Car3*, L5/6 NP, astrocytes, OPC, oligodendrocytes, endothelial cells, VLMC, and microglia‐PVM.

### 2.3 Cell composition analysis

Cell composition analyses of the ROS/MAP snRNA‐seq dataset include both cell fraction comparison and cell abundance comparison between sexes and AD diagnostic groups for the eight major cell types as well as for the 67 subgroups (unidentified “NA” [unassigned] subgroups were removed). Cell fractions (percentage) were calculated with “NA” subgroups included and compared by two‐way ANOVA with a sex‐by‐AD dementia diagnosis interaction term. Cell abundance analyses were carried out using the crumblr (version 0.99.11) R package (https://DiseaseNeurogenomics.github.io/crumblr). For cell abundance analyses, subpopulation cell counts were centered log ratio (CLR) transformed using the “crumblr” function, which also returned observation‐level inverse‐variance weights for use in weighted linear models to test the compositional change of each cell type separately, following the formula: ∼ age at death + *post mortem* interval (PMI) + sex + AD dementia diagnosis + sex × AD dementia diagnosis implemented by the “dream” function in the crumblr package. A total of 299 participants were included in this analysis after removing those without PMI or AD dementia diagnosis data. *p* values were adjusted using a Bonferroni correction across all eight cell types or 67 subgroups tested.

### 2.4 AD endophenotype measurements

For the ROS/MAP cohorts, global amyloid beta load (Aβ, “amyloid” variable from Research Resource Sharing Hub [RADC]) and paired helical filament tau tangle density (tau, “tangles” variable from RADC) were measured at autopsy by immunohistochemistry staining, quantified as the average percentage of area occupied by Aβ (using antibodies specific to Aβ42) or tau (using antibodies specific to AT8 epitope of abnormally phosphorylated tau) across eight brain regions at autopsy: hippocampus, angular gyrus, and the entorhinal, midfrontal, inferior temporal, calcarine, anterior cingulate, and superior frontal cortices. Values were then transformed to approximate a normal distribution.^46^

The global cognitive score was calculated as a composite measure of global cognitive function by converting raw scores from 17 cognitive tests to *z*‐scores and calculating their average.^47^ For cross‐sectional analyses of cognitive function, the global cognitive score at last visit before death was used. Due to the mixed‐effects model required for snRNA analysis, the longitudinal cognitive trajectory was evaluated by quantifying a cognitive slope derived by leveraging a linear mixed‐effects model with global cognition as the outcome, the intercept, and years from last cognitive visit entered as both fixed effects and random effects. The random slope is the estimated person‐specific rate of change in the global cognition variable over time. This person‐specific estimate was added to the fixed‐effects slope to calculate each individual's annual rate of change.

For the SEA‐AD DLPFC cohort, we used gold‐standard Consortium to Establish a Registry for Alzheimer's Disease (CERAD) neuritic plaque staging (none, sparse, moderate, and frequent) and Braak neurofibrillary tangle staging (stages 0–VI) and as semi‐quantitative outcomes represented Aβ and tau pathology, respectively, due to a lack of quantitative pathology scores during the preparation of this manuscript. Slopes of memory decline over time based on the original cognitive abilities screening instrument (CASI) scores (to derive stable and generalizable estimates) were calculated using mixed‐effects models with an unstructured covariance matrix, where time was parameterized as years before death. The CASI score at the last visit was used to represent global cognition at last visit.

### 2.5 Sex‐specific association analyses with AD endophenotypes

Gene expression associations using the ROS/MAP dataset with four AD endophenotypes (global cognition longitudinally and at last visit before death, Aβ, and tau at death) and AD dementia diagnosis were conducted for each gene in each of the eight major cell types using a negative binomial mixed model implemented in the Nebula R package (version 1.4.2).^48^ The number of genes analyzed in each association model ranged from 14,537 to 16,672. Only participants with a PMI < 24 h were included, which yielded 417 participants.

To estimate associations with Aβ, tau, global cognition at last visit before death, and AD dementia diagnosis, the following formula was used: gene count matrices ∼ AD endophenotype + sex + age at death + PMI. For analyses of longitudinal cognitive performance, years between last visit and death was added as a covariate, together with others mentioned above. Four participants who had >5 years between their last visit and death were removed from this analysis. The gene count matrices used in all association models were taken from the “count” slot of the SCT assay (the transformed RNA count assay generated by “sctransform” package mentioned above) from the Seurat object associated with each major cell type. All models were run in a combined sex and sex‐stratified manner. To estimate the sex‐interaction effect with each AD endophenotype, an interaction term, AD endophenotype × sex, was added to each model described above with the same covariates. Lowly expressed genes were removed from the analysis in Nebula by the default setting (counts per cell < 0.5% and number of cells with a positive count < 5) to obtain accurate estimates of the overdispersions. Finally, association models from Nebula with convergence scores less than or equal to −20 were removed from downstream analysis.

Sex‐specific association analysis of the SEA‐AD dataset was performed using the same model setup and inclusion criteria above. We carried out the association analyses in each of the predefined 25 cell subclasses (the L2/L3 subclass was split into two separate files for analysis due to computational limitation), and then reclassified them into seven major cell types to match ROS/MAP cell taxonomy for comparison. Only gene–phenotype association pairs that showed consistent direction of effect across all subclasses under the same major cell type were reserved for comparison.

Sex‐specific associations of ROS/MAP were defined as those that had nominal *p*‐values < 0.05 from sex‐interaction models and had false discovery rate (FDR)‐adjusted *p* values < 0.05 from sex‐stratified models and showed the same direction of effect on the same AD endophenotype in the same cell type tested.

### 2.6 Differential gene expression between sexes and AD dementia diagnosis

Differential gene expression analyses in each of the eight major cell types of the ROS/MAP dataset among participants with AD dementia or normal cognition were carried out using the Nebula R package, as described above, with the same covariates, that is, sex, age at death, and PMI. This analysis was set up to detect gene expression changes not only within the same sex cross diagnostic groups, but also between sexes within the same diagnostic group for completeness.

We used the final consensus cognitive diagnosis (“cogdx”^49^ variable from RADC) to categorize participants into clinical AD dementia group or normal cognition group. In total, 299 participants had cogdx scores, among which 142 with “cogdx” = 1 were assigned to the normal cognition group, and 157 participants with “cogdx” = 4 or “cogdx” = 5 were assigned to the clinical AD dementia group.

### 2.7 Gene set enrichment

The gene set enrichment analysis of gene lists generated from sex‐stratified association models was conducted using the “fgsea” function from the fgsea R package.^50^ We used 20,354 unique genes that were detected in all cell types in post‐QC ROS/MAP snRNA‐seq cohorts as the background set for testing to avoid bias. We ranked all genes from each sex, cell type, and AD phenotype combination by statistical significance using ranking values calculated by −log10 ({*p* value}) × sign ({log value of fold change, logFC}). Under this rank metric, positively associated (logFC > 0) genes with relatively small *p* values appear at the top of the list, and negatively associated (logFC > 0) genes with small *p* values at the bottom. We removed genes that have a ranking of infinity due to having very small *p* values. Then we tested each of the 64 pre‐ranked gene lists (four AD phenotypes × eight cell types × two sexes) against 50 signature gene sets in HALLMARK from MSigDB^51^ (v2023.1) with 1,000,000 permutations. *p* values of enrichment tests were adjusted using Bonferroni correction cross 3200 tests for HALLMARK gene sets.

### 2.8 Intercellular communication profiling

Intercellular communication patterns among seven major cell types (merging both excitatory neuron subgroups) were analyzed using the CellChat (version 2.0) R package^52^ with default setting, without projecting gene expression data onto human protein–protein interaction network. ANOVA (for more than two groups) and Wilcoxon rank‐sum (between two groups) tests were used for statistical comparison. Tukey's Honestly Significant Difference tests after ANOVA were run to detect differences between any two groups.

### 2.9 Gene ratio calculation

To examine whether sex‐specific genes located on sex chromosomes were enriched compared to those from autosomes, we calculated gene ratio by dividing the total annotated gene number on each chromosome, based on the Human Genome Assembly GRCh38.p14.^53^

### 2.10 Statistical analysis

Statistical analyses were conducted in RStudio (R version 4.3.1). Significance was set a priori to α = 0.05. *p* values from all sex‐stratified models were corrected for multiple comparisons using the FDR procedure across all 1,003,028 tests combining all four endophenotypes for ROS/MAP. Overlapping sex‐specific associations between SEA‐AD and ROS/MAP from sex‐stratified models of SEA‐AD were corrected for 2660 tests using the R function “p.adjust” with “BH” as the method.

## 3 RESULTS

To summarize, we defined an association as protective when higher gene expression was associated with a better AD‐related endophenotype (i.e., less neuropathology or better cognition performance) and an association as risk when higher gene expression was associated with a worse AD‐related endophenotype.

### 3.1 ROS/MAP cohort characteristics

Our discovery dataset, the post‐QC ROS/MAP snRNA‐seq dataset, was derived from 424 non‐Hispanic White donors, with a mean age at death of 89 years and an average of 16 years of education. In addition, 68% were females, 38% of participants had a final consensus cognitive diagnosis that met clinical AD dementia criteria, 16% carried at least one *APOE* ε2 allele, and 26% carried at least one *APOE* ε4 allele. Females and males differed statistically in their age at death, education, Aβ, and tau, with females presenting at an older age at death, with less education, and higher Aβ and tau than males (Figure 1A; Table S1). Compared to individuals with normal cognitive function, those with AD dementia had a higher age at death, higher Aβ and tau burden at autopsy, and worse cognitive function and included a greater proportion of *APOE* ε4 carriers (Figure 1A; Table S1).

### 3.2 Identifying sex‐ and disease‐dependent cell subpopulations

We first investigated whether sex, AD dementia, or their interaction could influence the composition of cell types to provide a foundation for our explorations into the sex‐specific transcript associations with AD endophenotypes. The ROS/MAP snRNA‐seq dataset was clustered into eight major cell types, which were further clustered and annotated into 67 subpopulations (Figure 1B). For eight major cell types, we observed no compositional differences by sex, AD dementia diagnosis, or sex‐by‐diagnosis interactions in both cell fraction and cell abundance (Figure 1C; Table S2–S3). For 67 cell subpopulations, seven subpopulations were sex‐dependent and four were AD dementia diagnosis‐dependent (Figure 1D–E; Figure S1). Females had a higher fraction of Oli.6 (enhanced‐translation subgroup), Oli.12, OPC.1 (enhanced‐mitophagy subgroup), and VIP inhibitory neurons, while males had a higher fraction of Oli.3, Oli.9, and SST inhibitory neurons (Figure 1D–E, Figure S1D). In AD cells, we observed higher fractions of disease‐elevated microglia and astrocytes as expected, together with lower fractions of arterial smooth muscle cells (aSMC or SMC.1) within the vascular niche and excitatory neuron subtype 3 (Exc.3, mapped to cortical layer L2 and L3 by the Allen Brain Map; Figure 1E, Figure S1C). Notably, no significant sex and AD dementia diagnosis interaction effect was observed, suggesting cell type alterations in sex or disease are largely shared across diagnostic groups or sexes, respectively (Table S2). Similar results were observed in cell abundance comparisons (Figure 1E; Figure S1A), and again no sex‐by‐AD dementia diagnosis interaction effect was observed (Table S3).

### 3.3 Identifying sex‐specific associations with AD endophenotypes

**FIGURE 2:**
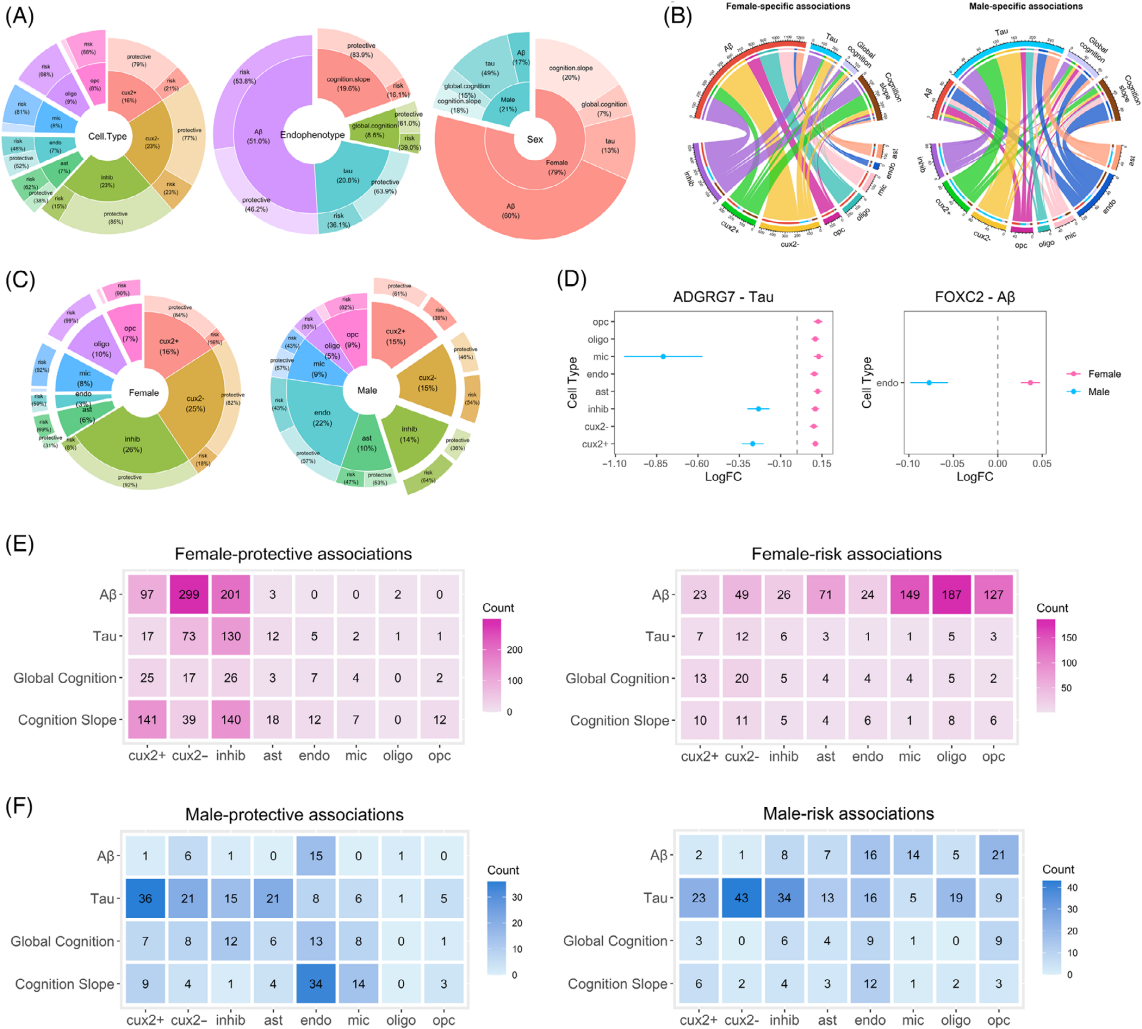
Sex‐specific gene associations with AD endophenotypes. (A) Pie‐donut chart showing relative proportions of sex‐specific associations (*N* = 2660) by sex, endophenotype, major cell types, and associations effect on four endophenotypes. (B) Chord diagram comparing absolute number of female‐ and male‐specific associations among eight major cell types and four endophenotypes. (C) Pie‐donut chart comparing percentage of female‐ and male‐specific associations within each cell type, together with percentage of protective and risk effect associations. (D) Forrest plot highlighting sex‐specific associations in same cell type with *ADGRG7* and *FOXC2* showing opposing effect between sexes for tau and Aβ, respectively. (E) Heatmap showing absolute number of female‐protective and female‐risk associations among eight major cell types and four endophenotypes. (F) Heatmap showing absolute number of male‐protective and male‐risk associations among eight major cell types and four endophenotypes. AD, Alzheimer's disease; ast, astrocytes; cux2+/cux2−, CUX2+ or CUX2− excitatory neurons; endo, endothelial cells; inhib, inhibitory neurons; LogFC, log2 fold change; mic, microglia; oligo, oligodendrocytes; opc, oligodendrocyte precursor cells.

We next investigated how gene expression in different brain cell types associated with common AD endophenotypes, including Aβ, tau, global cognitive performance at last visit, and cognitive decline (see “Methods” for definition). Among the four endophenotypes studied, we identified a total of 2660 sex‐specific associations (Figure 2A; Table S4). Interestingly, among the sex‐specific associations, there were 10 genes showed FDR significant associations with the same AD endophenotype in the same cell type within each sex: *ADGRG7*, *EYA1*, and *FOXC2* for Aβ; *ADGRG7*, *ASIP*, *GPCPD1*, *KCNMB3*, *MRAS*, *SLC44A1*, and *UQCC1* for tau; and *IFI44L* for cognitive trajectory (Table S5). Among these, two showed opposing association effect between sexes, including *FOXC2* in endothelial cells with Aβ, and *ADGRG7* in three cell types (CUX2+ excitatory neurons, inhibitory neurons, and microglia) with tau, with all showing risk effects in females but protective effects in males (Figure 2D; Table S5).

Among the female‐specific associations we identified, 77% of the risk associations were from glial cells, while 93% of protective associations were from neurons (Figure 2B–C, E). Within neuronal associations, 87% showed a protective effect. Among males, endothelial cells showed many male‐specific associations with all AD outcomes (Figure 2B–C,F), a much more prominent role that did not appear in females. In contrast to the female‐specific associations outlined above, we did not see a striking split between neuronal and glial cell types regarding protective and risk effects.

### 3.4 Replication of sex‐specific associations with AD endophenotypes

**FIGURE 3:**
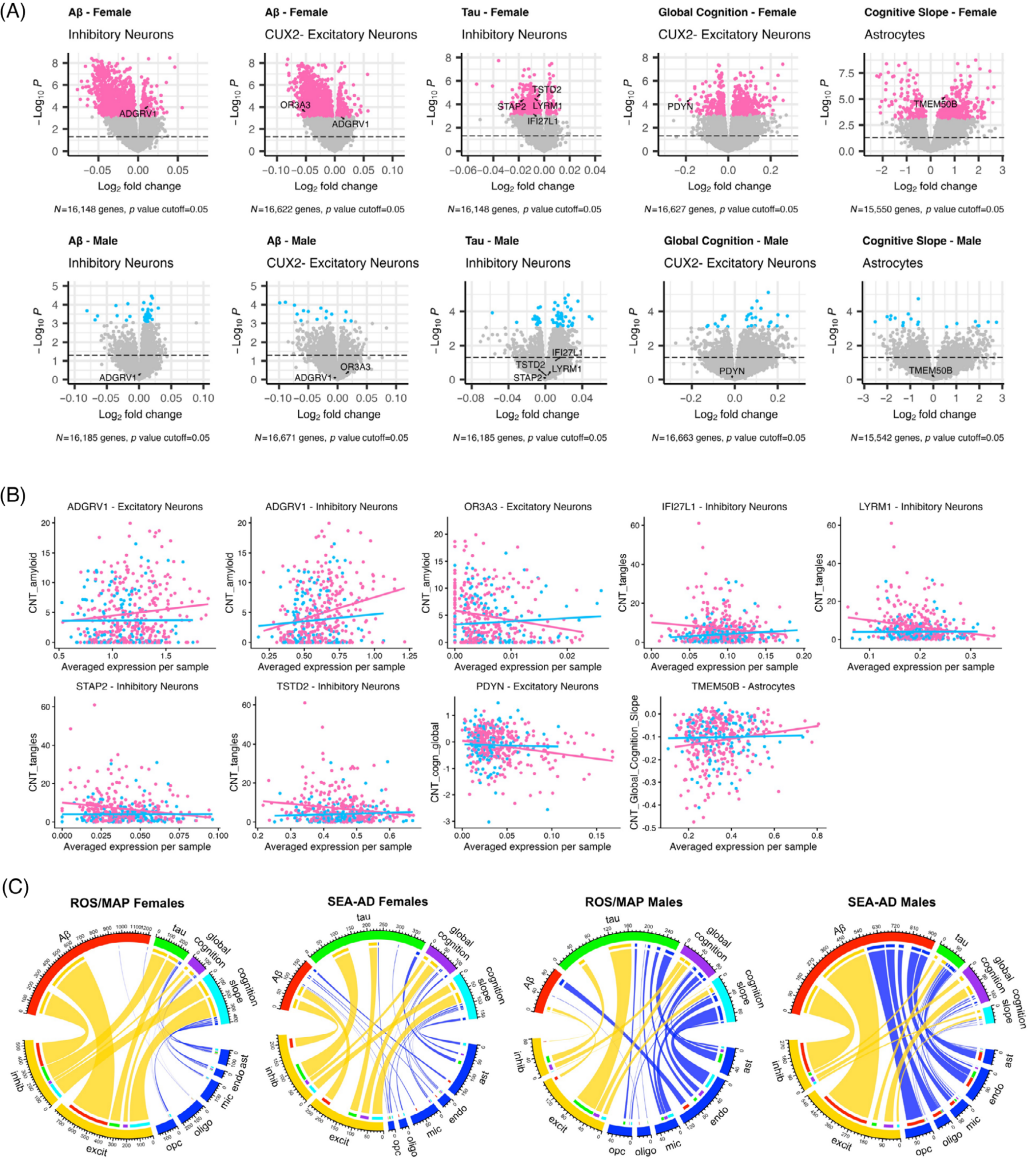
Replication of ROS/MAP sex‐specific gene associations in SEA‐AD. (A) Volcano plot showing log2 fold change of gene expression associated with one unit increase of annotated endophenotype in each sex. Pink dots represent FDR associations in females; blue dots represent FDR associations in males; gray dots represent non‐FDR results. (B) Scatter plot showing correlation of highlighted gene expression and the annotated endophenotype in specific cell type. Pink dots represent female samples; blue dots represent male samples. Outliers beyond 4 standard deviations were removed from scatter plots. Lines in scatter plots represent unadjusted linear fit. (C) Chord diagrams showing sex‐specific associations in females and males from ROS/MAP and SEA‐AD cohorts. The length of each sector of the circle represents the total number of associations. Only protective links are displayed in the plots. Cell types were color‐coded to show difference between neurons and glial cell types. ast, astrocytes; excit, excitatory neurons; endo, endothelial cells; FDR, false discovery rate; inhib, inhibitory neurons; mic, microglia; oligo, oligodendrocytes; opc, oligodendrocyte precursor cells; ROS/MAP, Religious Orders Study and the Rush Memory and Aging Project; SEA‐AD, Seattle Alzheimer's Disease Brain Cell Atlas.

To validate our sex‐specific associations, we carried out the same analysis using snRNA‐seq data derived from the same brain region of donors from a completely independent cohort called SEA‐AD.^42^ Despite differences in cohort characteristics, sequencing data generation, quality control methods, and cell type taxonomies, we successfully replicated nine novel female‐specific associations involving eight unique genes in the SEA‐AD dataset, including *ADGRV1* and *OR3A3* with Aβ; *IFI27L1*, *LYRM1*, *STAP2*, and *TSTD2* with tau; *PDYN* with global cognition; and *TMEM50B* with cognitive trajectory (Figure 3A,B). *ADGRV1*, *OR3A3*, and *PDYN* showed associations in excitatory neurons; *ADGRV1*, *TSTD2*, *IFI27L1*, *LYRM1*, and *STAP2* in inhibitory neurons; and *TMEM50B* in astrocytes. Interestingly, we also replicated the pattern of sex‐specific effects in AD across cell types and endophenotypes, such that protective associations for females were enriched in neurons (93% for ROS/MAP, 80% for SEA‐AD), whereas those for males were evenly distributed across cell types (Figure 3C). In addition, among all glial cell types, endothelial cells contributed many male‐specific associations across datasets (40% for ROS/MAP, 31% for SEA‐AD; Figure 3C).

### 3.5 Differential expression and sex chromosome representation of sex‐specific associations

**FIGURE 4:**
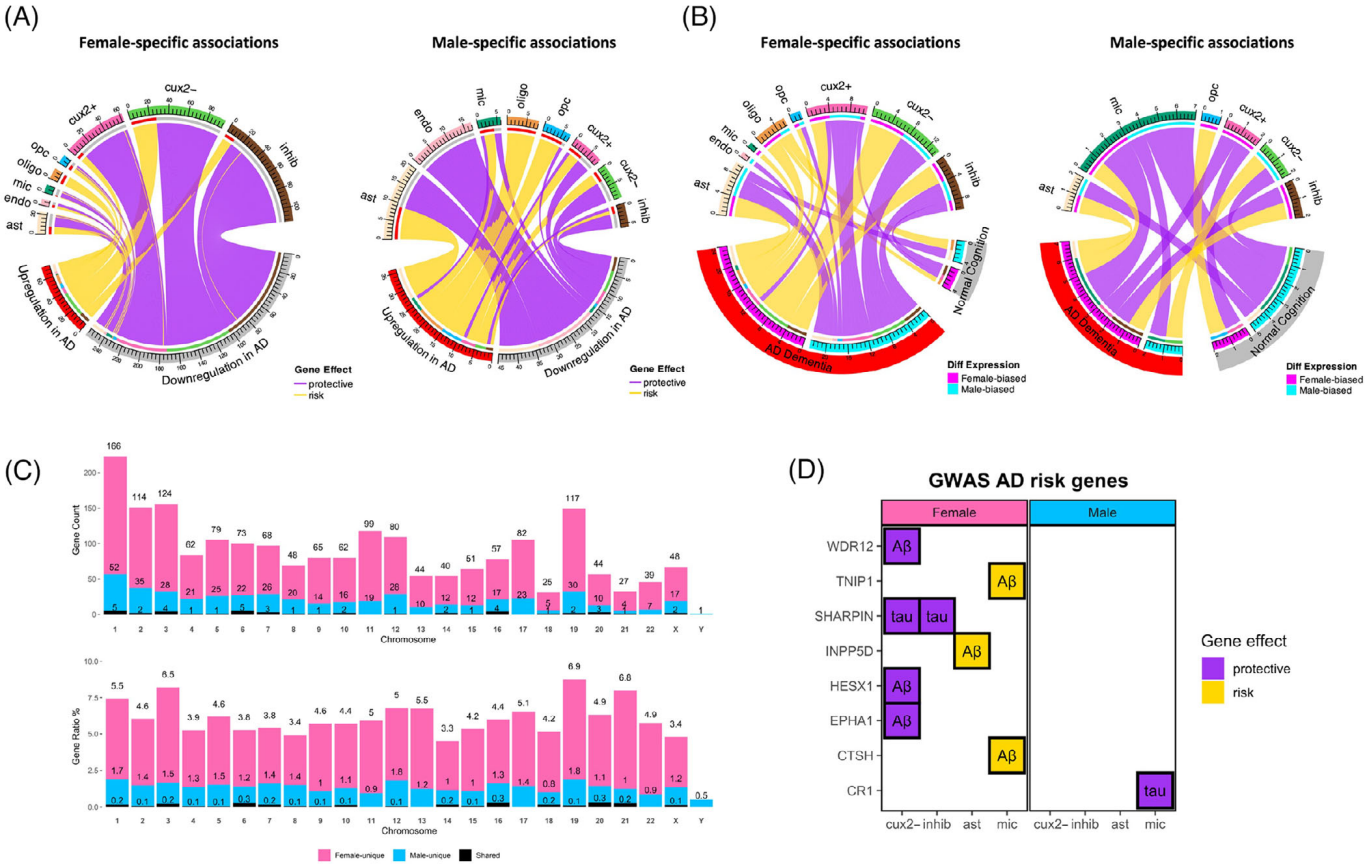
Additional characterizations of sex‐specific effect genes. (A) Chord diagram showing sex‐specific association genes that are differentially expressed among those with AD dementia. (B) Chord diagram showing sex‐specific association genes that are differentially expressed between sexes. Purple links represent protective sex‐specific associations; yellow links represent risk sex‐specific associations. (C) Absolute count and ratio of sex‐specific association genes on each chromosome. Total annotated gene number of each chromosome was extracted based on Human Genome Assembly GRCh38.p14. Female‐unique represents genes that only show sex‐specific associations among females, male‐unique represents genes that only show sex‐specific associations among males, shared represents genes that show sex‐specific associations with both sexes. (D) Eight sex‐specific effect genes that also have been identified as AD risk genes from GWAS. Gene effect indicates the directionality of sex‐specific associations with each annotated AD endophenotype. AD, Alzheimer's disease; ast, astrocytes; cux2+/cux2−, CUX2+ or CUX2− excitatory neurons; DE, differential expression; endo, endothelial cells; GWAS, genome‐wide association study; inhib, inhibitory neurons; mic, microglia; oligo, oligodendrocytes; opc, oligodendrocyte precursor cells.

We then evaluated whether the sex‐specific associations were primarily driven by genes that are differentially expressed by sex or among those with AD dementia. Surprisingly, only 4% of sex‐specific gene–cell type combinations showed differential expression (DE) patterns in AD dementia (Figure 4A; Table S6), and only 1% were differentially expressed between sexes when stratified by diagnosis (Figure 4B; Table S7). Notably, among the nine replicated sex‐specific associations, two gene–cell type combinations showed DE among those with AD dementia regardless of sex; *PDYN* had an increased expression in CUX2− excitatory neurons, whereas *TMEM50B* had a decreased expression in astrocytes. In addition, both *ADGRV1* (in both CUX2− excitatory and inhibitory neurons) and *PDYN* (CUX2− excitatory neurons) showed female‐biased expressions among those with AD dementia.

Sex chromosome‐located genes can have physiological sex‐biased expression due to tissue‐dependent X chromosome inactivation (XCI)^54^ and escaping^55^, ^56^ processes in females or due to exclusive Y chromosomal gene expression in males. Closer examination revealed that 35 of the genes that showed sex‐specific associations with AD endophenotypes are known to be subject to XCI (Table S4), while three are known XCI escapees (*STS*, *SYAP1*, and *TXLNG*), four are classified as variable escape genes (*TSIX*, *TRPC5*, *DGAT2L6*, and *FOXO4*), and one is in the pseudoautosomal region (*ASMTL‐AS1*) and is expected to escape XCI.^57^ Further examination of the sex‐specific associations located on the X‐chromosome in our dataset revealed seven that showed sex‐biased expression in the cell type on which they exerted their sex‐specific effect, including *BX842570.1* (inhibitory neuron), *DDX3Y* (microglia), *STS* (CUX2+ excitatory neuron), *TSIX* (CUX2+ excitatory neuron), *TXLNG* (oligodendrocyte), *ASMTL‐AS1* (CUX2– excitatory neuron), and *SYAP1* (oligodendrocytes). *DDX3Y* is the only Y chromosomal gene on our list and it showed male‐biased expression as expected.

Additionally, among the sex‐specific associations, 67 genes (3%) were on the X chromosome and only one was on Y chromosome (Table S4). We did not see an over‐representation of genes from sex chromosomes relative to the autosomes either by the absolute count or by ratio (Figure 4C). None of the eight replicated sex‐specific effect genes was located on sex chromosomes either.

### 3.6 Eight sex‐specific association genes were known AD risk genes from genome‐wide association studies (GWAS)

To further support the important role these sex‐specific gene associations might play in AD pathogenesis, we explored whether any had been previously implicated in the genetic architecture of AD based on published GWASs of AD. We examined 112 recently reviewed AD risk loci from GWASs^58^ and found eight of our sex‐specific effect were identified as AD risk genes, including *CR1*, *WDR12*, *INPP5D*, *HESX1*, *TNIP1*, *EPHA1*, *SHARPIN*, and *CTSH* (Figure 4D). All these genes showed sex‐specific associations with either Aβ or tau pathology. *CR1* showed a male‐specific effect, while the rest showed female‐specific effects. Higher levels of *CR1* in microglia were associated with less tau among males. For the female‐specific associations, higher levels of four genes in neurons were associated with better AD endophenotypes, including *EPHA1*, *HESX1*, and *WDR12* in CUX2− excitatory neurons with Aβ, and *SHARPIN* in both CUX2− excitatory and inhibitory neurons with tau. In contrast, among the glial cells, higher *INPP5D* in astrocytes, and *CTSH* and *TNIP1* in microglial was related to a higher Aβ. Sex‐specific associations for the lead variants at these candidate gene loci have not been reported in the literature.

### 3.7 Sex‐stratified association genes are enriched for immune‐, inflammatory‐, damage‐related, stress‐response pathways

**FIGURE 5:**
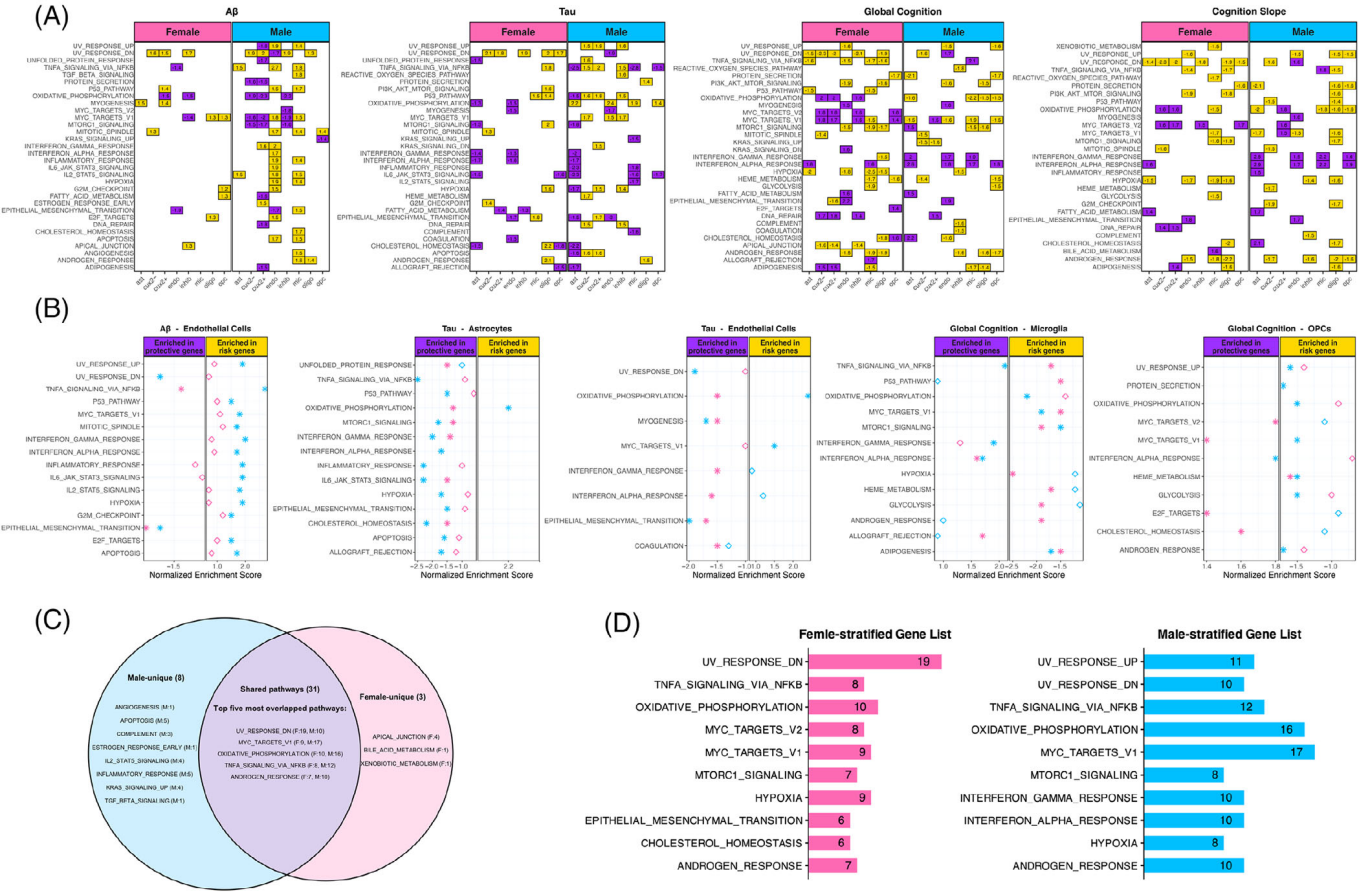
Gene set enrichment pattern of sex‐stratified AD endophenotype associations. (A) All global FDR < 0.05 gene set enrichment results from sex‐stratified associations for each of four endophenotypes tested. Purple tiles indicate risk association genes enriched; yellow tiles indicate protective association genes enriched. The annotated number in each tile is normalized enrichment scores extracted from the analysis. (B) Five combinations of endophenotype and cell type that showed enrichment of the same pathway in opposite directions across sexes (pink represents female, sky‐blue for male). Solid data points indicate FDR enrichment, hollow data points represent non‐FDR enrichment. (C) Comparison of FDR‐enriched pathways between sexes. We list all sex‐unique enriched pathways but only present the top five (those enriched in the most cell type‐endophenotype combinations) shared pathways for brevity. F, females; M, males. (D) Top 10 FDR enrichment pathways for each sex. The annotated number in each bar represents the frequency that each individual pathway was FDR‐enriched across all cell type‐endophenotype combinations. AD, Alzheimer's disease; ast, astrocytes; cux2+/cux2−, CUX2+, or CUX2− excitatory neurons; endo, endothelial cells; FDR, false discovery rate; inhib, inhibitory neurons; mic, microglia; oligo, oligodendrocytes; opc, oligodendrocyte precursor cells.

To investigate sex differences in the molecular pathways implicated in AD, we performed the gene set enrichment analyses leveraging sex‐stratified results from each endophenotype and cell type. Numerous biological pathways showed a sex‐specific enrichment pattern in various cell types, many are immune‐, inflammatory‐, damage‐related, and stress‐response pathways (Figure 5A; Table S8). Irrespective of cell type, we observed many functional pathways enriched in both sexes (Figure 5C–D), including 22 unique pathways that were enriched in the same cell type with the same endophenotype in the same direction (Table S8). Meanwhile, we also observed many pathways that were uniquely enriched in one sex, including angiogenesis, apoptosis, complement, early estrogen response, and several immune and inflammatory pathways in males, including apical junction, bile acid metabolism, and xenobiotic metabolism in females (Figure 5C). Among the enriched pathways, we saw multiple immune/inflammatory pathways enriched for risk effect genes associated with Aβ in endothelial cells among men and MYC targets V1 and V2 pathways enriched for protective effect genes associated with cognitive function in neurons among females (Figure 5A). In these pathways, we found several sex‐specific effect genes that appeared to drive the enrichment along with other genes, including *CXCL10*, *CXCL11*, *FOSB*, and *RHOB* in endothelial cells in men and *AIMP2* and *MRTO4* in neurons in females.

Most interestingly, we observed three pathways that were enriched for the same cell type–endophenotype combination but with the opposite effect genes dominating the leading edge (i.e., the “core” group of genes that contribute the most to the enrichment score) by sex (Figure 5B). The first one was TNFα signaling via NF‐κB, which was enriched for both microglial gene associations with global cognition and endothelial gene associations with Aβ. In both cases, the pathway was enriched with protective effect genes among males, but risk effect genes among females. The second pathway that showed a sex‐dimorphic pattern was oxidative phosphorylation (OXPHOS) in both astrocytes and endothelial cells with tau, and the third was MYC targets V1 in OPCs with global cognition. In the OXPHOS and MYC target V1 cases, protective effect genes drove the enrichment score among females, but risk‐effect genes among males.

### 3.8 Sex‐specific effects led to sex differences in intercellular communication in AD brain

**FIGURE 6:**
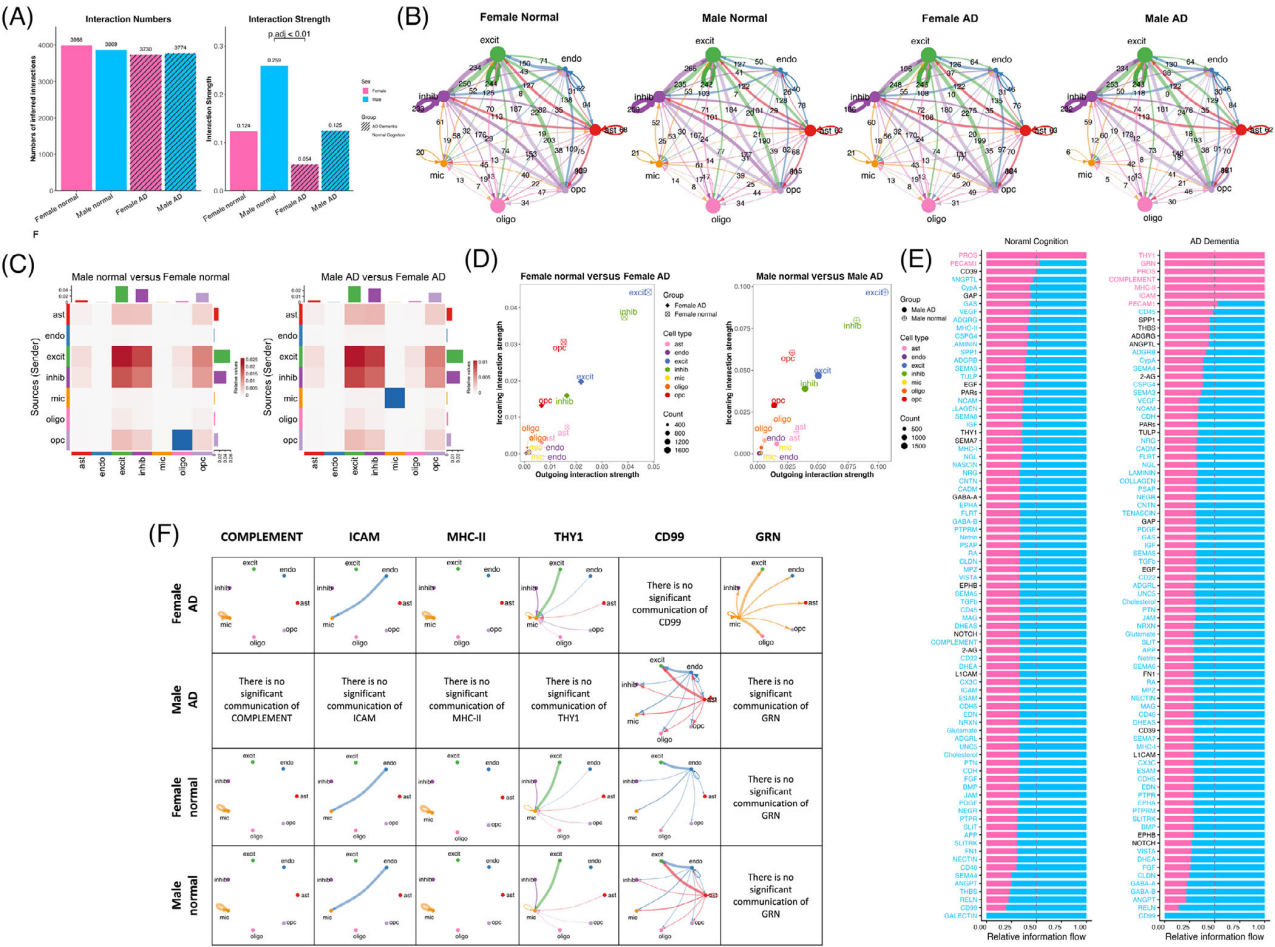
Intercellular communication comparison between sex and AD dementia diagnostic groups. (A) Comparison of total count and strength of all curated intercellular communication interactions for four groups. (B) Overall information flow of four groups. Annotated numbers showing total count of interactions between each cell type pair. (C) Differential interaction strength heatmap between sexes among those with normal cognition or AD dementia. The colored bar plot at the top represents the sum of incoming signaling displayed in the heatmap. The colored bar plot on the right represents the sum of outgoing signaling. The bar height indicates the degree of change in terms of the number of interactions or interaction strength between sexes. In the heatmap, red (or blue) represents increased (or decreased) signaling in the male dataset compared to the female dataset. (D) Changes in outgoing and incoming interaction strength for each major cell type in each sex with or without AD dementia. (E) Comparison of information flow between sexes for each signaling pathway among each diagnostic group. Each inferred signaling network is defined by the sum of the communication probability (strength) among all pairs of cell groups in the inferred network. Significant signaling pathways were ranked based on differences in the overall information flow within the inferred networks between sexes. A paired Wilcoxon test is performed to determine whether there is a significant difference in the signaling information flow. Signaling pathways colored pink are enriched in females; those colored blue are enriched in males; and those colored black show no difference between sexes. (F) Circle plot of intercellular communications of COMPLEMENT, ICAM, MHC‐II, THY1, CD99, and GRN networks in four groups. Circle sizes representing major cell type are proportional to the number of cells in each cell group. The width of chords represents the strength of the total communication. AD, Alzheimer's disease; ast, astrocytes; excit, excitatory neurons; endo, endothelial cells; inhib, inhibitory neurons; mic, microglia; oligo, oligodendrocytes; opc, oligodendrocyte precursor cells.

Regulation of chemical‐based signal transmission among brain cells is crucial for maintaining the health and function of the central nervous system (CNS). We were interested in investigating whether the sex‐specific associations led to alterations in such cell–cell signaling by comparing the intercellular communication patterns observed across sex and disease. First, on a global level, all four groups (females with AD dementia, males with AD dementia, females with normal cognition, and males with normal cognition) had comparable cell–cell communication count and strength (probability), except for a significant reduction in communication strength among females with AD dementia compared to males with normal cognition (Figure 6A). The majority of intercellular communication signals were among neurons, astrocytes, and OPCs for all groups (Figure 6B). Among participants with normal cognition, the only communication strength that was inferred to be stronger in females compared to males was from OPCs to oligodendrocytes, but the difference shifted to a stronger communication strength within microglia among females compared to males with AD dementia (Figure 6C). For both sexes, the biggest reduction in cell–cell communication in AD dementia was observed among excitatory neurons, inhibitory neurons, and OPCs (Figure 6D).

Second, for individual cell–cell communication signals across cell types, several showed sex‐specific alterations in AD dementia (Figure 6E). For example, compared to AD females, AD males lacked meaningful inferred communication of the THY1, COMPLEMENT, MHC‐II, and ICAM signals, due to significant downregulation of these pathways among those with AD dementia. Interestingly, most of the altered signals are incoming signals to microglia that were present in males with normal cognition, except for the endothelial‐sent THY1 signal that was unique to females (Figure 6F). Conversely, AD females lacked meaningful communication signals of CD99 compared to AD males, due to a significant reduction in this cell communication pathway among females with AD dementia (Figure 6F). CD99 was sent by both endothelial cells and astrocytes in males regardless of diagnosis; however, females with normal cognition only had endothelial‐sent CD99, indicating the astrocyte CD99 signaling was unique to males. In addition, AD females showed a unique signal from microglia to every cell type including itself, namely, *GRN*, which showed no inferred communication among all other groups (Figure 6F). This unique microglial GRN signal among females with AD dementia seems to reflect an increase in microglial *GRN* expression among females with AD dementia.

Third, when we conducted a pairwise group comparison of individual signals accounting for whether a particular cell type was sending or receiving signals, we found a total of 34 group‐specific signals, two‐thirds of which were specific to either females with AD dementia (32%) or males with normal cognition (33%) (Table S9). This observation indicated downregulation of male‐specific intercellular communication in males with AD dementia and upregulation of AD‐specific signals in females with AD dementia as the primary mechanism underlying sex‐specific cell signaling alterations. In addition, near one‐third of these group‐specific communications involved microglia, hinting at its leading role in driving sex‐specific cell communication alterations in AD. Among 34 group‐specific signals, we performed a more detailed investigation into 19 of them that involved at least one gene with a sex‐specific association with any AD endophenotype. Six out of the 19 group‐specific signals showed intercellular communication alterations that aligned with the direction of effect and the cell type implicated in our sex‐specific gene association analyses above. These altered signals are COLLAGEN, FN1, JAM, LAMININ, SPP1, and TENASCIN, all of which contribute to unique incoming signals to microglia among females with AD dementia (Figure S2; Table S9). It should be noted that each of these signals also presented with signals coming from other cell types, but the incoming signal to microglia was the most notable sex difference in AD dementia (Figure S2).

**FIGURE 7:**
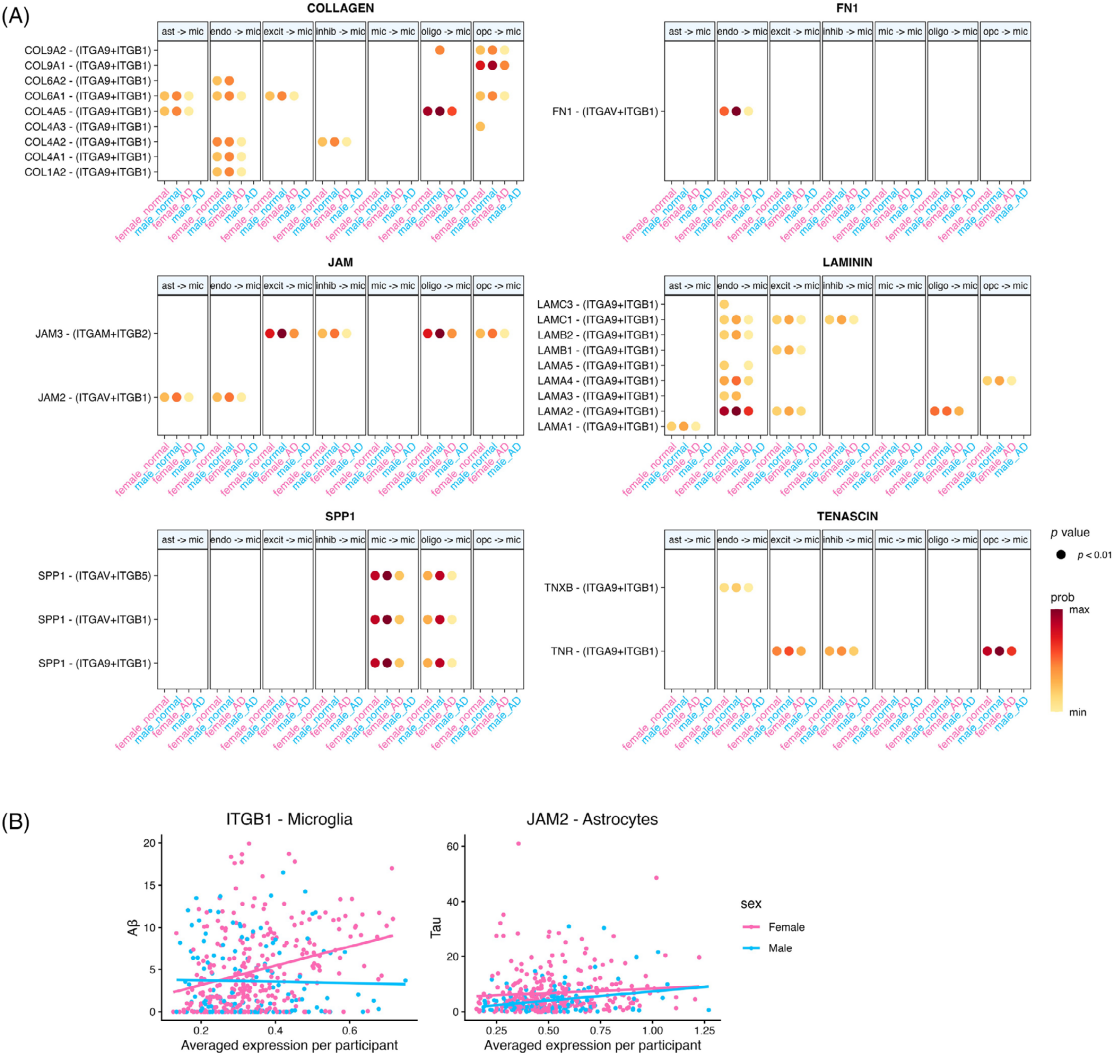
ITGB1 and *JAM2* induced six sex‐unique microglial‐incoming signals under AD dementia. (A) Dot plot showing all enriched intercellular communication (L‐R pair interactions) from every major cell group to microglia, evaluated by sex and diagnosis. (B) Scatter plot showing correlation of *ITGB1* expression with Aβ in microglia and *JAM2* expression with tau in astrocytes. Pink dots represent female samples; blue dots represent male samples. Outliers beyond 4 standard deviations were removed from scatter plots. Lines in scatter plots represent unadjusted linear fit. AD, Alzheimer's disease.

The most interesting fact about these six signals are that they share a common receptor on microglia, ITGB1 (encoded by *ITGB1*). In all cases, the sex difference in this signaling pathway was driven by a more severe downregulation of incoming ITGB1 signals to microglia among males with AD such that there were no inferred communication signals in this group (Figure 7A). Microglial *ITGB1* indeed showed more expression in females with AD compared to males with AD (*p* = 0.02, FDR = 0.4; Table S7), but no sex‐by‐AD interaction effect was observed (*p* = 0.06). Notably, *ITGB1* in microglia shows a risk association with Aβ among females (FDR = 0.01; Figure 7B), suggesting these six microglial incoming signals might play a role in sex‐specific risk for Aβ accumulation. Furthermore, in addition to using ITGB1 as a receptor, one of the six altered signals named JAM also involves another sex‐specific effect gene *JAM2*, which encodes one of its ligands by the same name (JAM2). The JAM2‐(*ITGAV+ITGB1*) interaction induced a unique incoming signal to microglia from astrocytes and endothelial cells in AD females (Figure 7A). *JAM2* expression in astrocytes was significantly increased (FDR = 0.03) in AD dementia compared to normal cognition in both sexes (female *p* = 0.004, male *p* = 0.09), but it did not show sex‐biased expression in any cell type regardless of disease diagnosis. Hence, the lack of JAM2‐(*ITGAV+ITGB1*) signal to microglia in AD males was due to the downregulation of *ITGB1*, as we discussed above. Since *JAM2* is a male‐specific risk gene in astrocytes for tau (FDR = 0.02; Figure 7B), we hypothesize that the JAM2‐induced *JAM* signal from astrocytes to microglia functions as a protective mechanism against tau pathology for men. The last sex‐specific effect gene that was involved in these six signals is *TNC*, which encoded a ligand for TENASCIN signals. However, TNC‐induced *TENASCIN* signal was not enriched in any group we analyzed.

## 4 DISCUSSION

**FIGURE 8:**
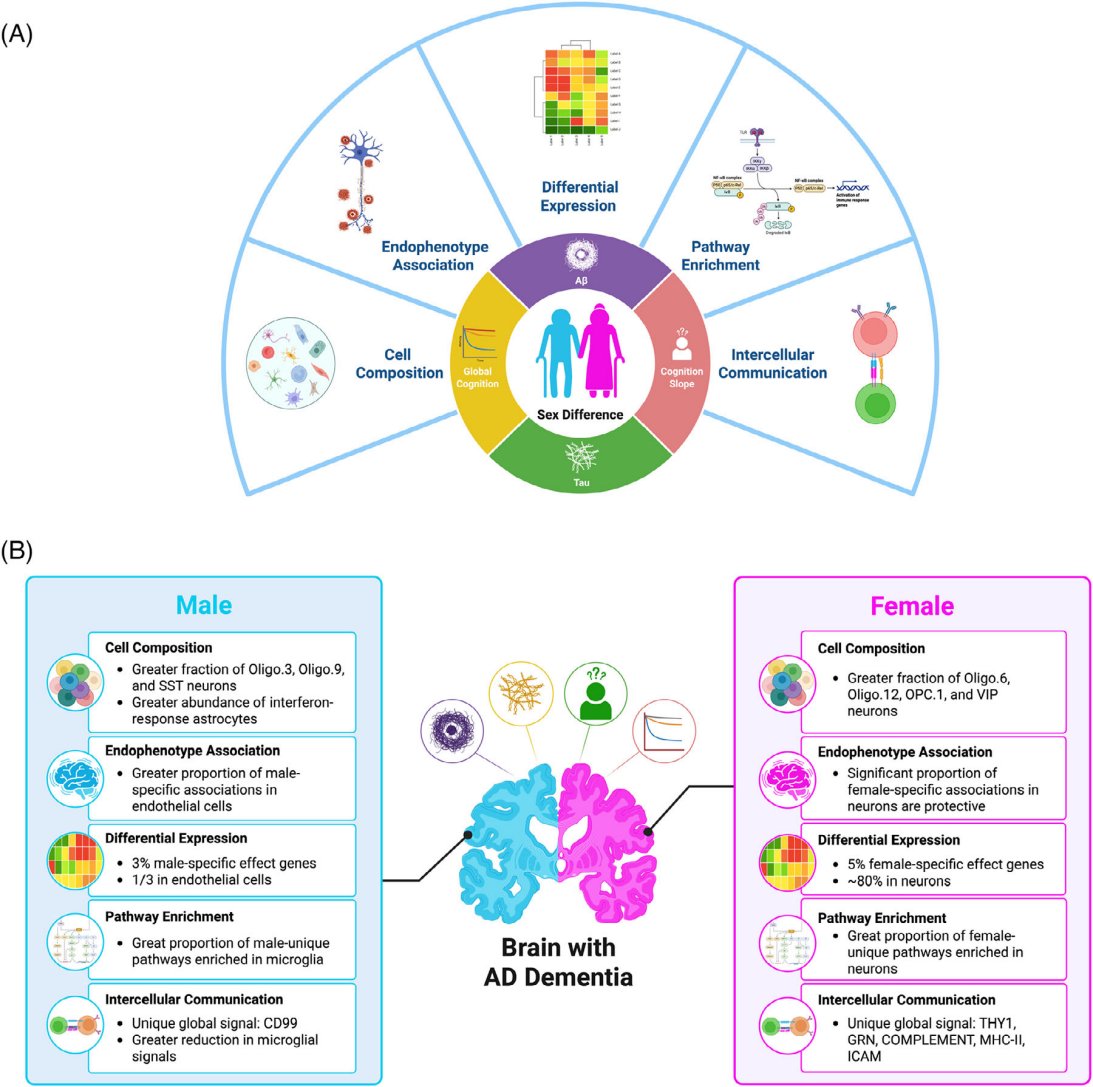
Summary of single‐cell landscape of sex‐specific differences in AD dementia. (A) Schematic illustration of analysis strategy. (B) Key sex‐specific associations with AD endophenotypes at single‐cell resolution. Both figures were created in BioRender. AD, Alzheimer's disease.

We have provided the most comprehensive assessment of sex‐specific transcriptomic associations with AD neuropathology and cognitive decline at a cellular resolution (Figure 8A). We have presented strong evidence of sex‐specific transcriptomic associations with AD endophenotypes and have provided a host of exciting sex‐specific targets for intervention that can be validated and prioritized in future mechanistic studies.

First, we highlighted a striking dissociation between protective and risk gene associations among females, with protective associations arising primarily from neurons (Figure 8B). These female‐specific associations may reflect a particular sensitivity of neuronal and glial cells to amyloidosis among females. Such a sex difference aligns with previous literature suggesting Aβ‐positive females are more susceptible to hippocampal atrophy and cognitive decline^59^ and a similar pattern in mouse models of brain amyloidosis.^60^ In contrast, we observed a male‐specific enrichment for endothelial cell associations, suggesting the vasculature may be particularly relevant to AD pathogenesis among males. It is known that sex hormones influence endothelial cell health and function, and hence there are notable differences in endothelial cell structure and function between the sexes.^61^ Endothelial cells have a sex‐dependent secretome^62^ and 14% to 25% of the endothelial transcriptome is sex‐biased, due to differences both at birth and acquired over a lifetime.^63^

Next, we identified and replicated nine novel female‐specific gene associations with AD endophenotypes, highlighting eight unique genes that may have therapeutic potential in women and have not been implicated in AD previously. In excitatory neurons, both *ADGRV1* and *OR3A3* encode proteins that are members of G protein‐coupled receptors,^64^ and both play a critical role in sensory development. ADGRV1 signaling is critical for the development of hearing and vision,^65^ and *OR3A3* encodes an olfactory receptor protein. In the case of olfaction, females show a faster rate of neuronal loss in the olfactory cortex during AD compared to males^66^; in addition, there is a tighter coupling between olfaction impairment and cognitive impairment among females compared to males.^67^ Interestingly, one of our replicated association genes in inhibitory neurons, *TSTD2*, is also implicated in a rare hearing‐loss disorder called camptodactyly‐tall stature‐scoliosis‐hearing loss syndrome.

Beyond the sensory effects, we also observed associations in inhibitory neurons among two genes involved in immune response: *IFI27L1* is part of the interferon‐stimulated gene family, which is typically involved in the immune response to viral infections and other inflammatory processes, and *STAP2* encodes an adaptor protein to enhance T‐cell‐receptor signaling,^68^ dysregulation of which can either cause immunodeficiency or autoimmunity linked to diseases such as multiple sclerosis.^69^ The final gene in inhibitory neurons, *LYRM1*, has been implicated in obesity‐associated insulin resistance^70^ and mitochondrial impairment.^71^

Two additional genes did not cluster with the others. In excitatory neurons, *PDYN* encodes the dynorphin neuropeptides, and polymorphisms and structural variants within this gene locus were reported to be associated with drug addiction.^72^ Interestingly, many *PDYN* variants exclusively showed associations with risk in female opioid addicts,^73^ suggesting opioids may indeed confer particular susceptibility in the female brain. In astrocytes, *TMEM50B* encodes a membrane protein that presents on the endoplasmic reticulum and Golgi apparatus. Located on chromosome 21, *TMEM50B* has been implicated in the neurophenotypes of Down syndrome due to being one of a few genes upregulated in model systems of Down syndrome, particularly in the cerebellum during development.^74^

Furthermore, we identified a host of sex‐specific biological pathways and cell–cell communication alterations that behave in a cell‐type‐specific manner (Figure 8B). We observed sex‐specific enrichment for a number of immune‐, inflammatory‐, damage‐related, and stress‐response pathways, including hypoxia, DNA repair, OXPHOS, and UV response, all of which are related to well‐known AD risk factors,^75^ with some enriched in opposite directions across sexes, such as TNF‐α signaling via NF‐κB, MYC targets, and OXPHOS pathways. Our intercellular communication profiling also pinpointed several immune and inflammatory response‐related pathways that are differentially dysregulated in AD between sexes, including THY1, COMPLEMENT, MHC‐II, ICAM, and CD99. Indeed, stress‐response gene signatures are upregulated in the AD brain, especially in late stages of disease,^75^, ^76^, ^77^, ^78^ and differences in both metabolism and stress‐response have been highlighted as key contributors to sex differences in AD in a recent review.^79^

We also highlighted the importance of microglia‐specific signals in contributing to sex differences in AD by first showing that 30% of the sex‐specific intercellular communication involves microglia. Microglia are the primary resident immune cells of the brain and have been increasingly recognized as a key player in sexual dimorphism in AD.^80^, ^81^ For example, distinct microglia‐specific immunometabolism patterns between sexes in AD in both immune and metabolic pathways was identified from a multi‐omics study.^82^ Furthermore, we nominated several microglia‐incoming signals through ITGB1 interaction as high‐quality candidates for a sex‐biased role in Aβ accumulation in AD that deserves further functional validation. *ITGB1* encodes the β1 subunit of integrin receptors and was identified as one of the five hub genes associated with the progression of AD and major depressive disorder.^83^ Most interestingly, microglial ITGB1 is a key component of the cell surface receptor system that mediates interaction between fibrillar Aβ (fAβ) and microglia to promote the clearance and phagocytosis of fAβ.^84^ While fAβ can induce overexpression of *ITGB1* transcription, the more potent neurotoxicity form Aβ oligomers can downregulate *ITGB1*.^85^ Our evidence that microglial *ITGB1* may act as a female‐specific risk factor for Aβ suggests that there may be a higher sensitivity of *ITGB1* to Aβ pathology in the female brain. In addition, the female‐specific incoming JAM signal from astrocytes to microglia provides an important insight and additional evidence of the immune interplay between astrocytes and microglia in AD.^86^ *JAM2* expression in astrocytes was identified as an important regulator of CD8 T‐cell migration into the CNS parenchyma during neuroinflammation.^87^

While there is emerging evidence of sex‐biased expression of genes on the sex chromosomes, particularly the X chromosome, contributing to AD pathogenesis,^79^, ^88^, ^89^ we did not observe a substantial enrichment for sex‐specific gene associations with AD endophenotypes on the sex chromosomes, suggesting that sex‐biased expression on the X and Y chromosomes may not be a major contributor to sex‐specific risk and resilience in AD. The most interesting finding among X‐chromosomal sex‐specific genes may be that some known XCI escapee genes appeared to change in their inactivation with disease. For example, *STS* in CUX2+ neurons only showed female‐biased expression among those with normal cognition, while *SYAP1* in oligodendrocytes only showed female‐biased expression among those with AD dementia. A very recent study^90^ revealed that aging remodeled transcription of the inactive X (Xi) and active X (Xa) across hippocampal cell types in female mice. Indeed, aging appears to change which genes escape inactivation in a cell‐specific manner, and our results suggest that such changes may also occur with disease in a cell‐specific manner. However, since the transcriptomic data we investigated did not provide direct evidence of the parent‐of‐X origin, we recognize the limitations of interpreting sex chromosome related findings discussed above.

Finally, our study identified a few sex‐ and disease‐dependent cell subpopulations, including several within inhibitory neurons and oligodendrocytes, matching a recent discovery of oligodendrocyte subpopulations that are particularly vulnerable in AD.^38^ We also identified two new disease‐dependent cell subpopulations in DLPFC, including aSMC and an L2 and L3 excitatory neuron population (Exc.3). A known AD risk variant, rs5011436A, is negatively associated with Exc.3 fraction^43^; however, more evidence is needed to understand the role of these subpopulations in AD pathogenesis.

This study had numerous strengths. The large sample size, deep cognitive and neuropathological phenotyping, and comprehensive replication dataset provided an unparalleled opportunity to explore sex differences in cell‐specific alterations in the AD brain. That said, our study also had many limitations. Both our discovery and replication cohorts had more female donors than male donors (female/male ratio is 2.1 for ROS/MAP and 1.5 for SEA‐AD), providing more statistical power in analyses of females and an unbalanced design for sex comparisons. In addition, both cohorts were limited to non‐Hispanic White individuals, limiting our generalizability to other populations. Our future effort will also include exploring more brain tissues, since AD pathogenesis involves multiple brain regions and there are variations in AD pathology both spatially and temporally among different regions.^91^ While we were able to replicate some effects, differences in sample size and cohort characteristics may have prevented additional replication. More effort in the field should focus on data integration^38^ and phenotype harmonization^92^ to tackle these critical issues. Furthermore, our approach of screening sex difference associations by a mixed use of *p* values from sex interaction models and FDR values from sex stratification models could increase type I error, though validating our initial screening findings in an independent cohort should diminish this limitation. Finally, summarizing gene associations as “protective” or “risk” has obvious shortcomings and could easily be misinterpreted. A causal relationship between the functional effect of an altered gene needs to be formally evaluated in mechanistic studies using in vitro or in vivo models to draw definitive conclusions about risk or protection.

## 5 CONCLUSION

This manuscript has provided the most comprehensive analysis of sex‐specific transcriptomic associations with AD endophenotypes at a cellular resolution. Due to differences in sample size, we had more statistical power to identify true female‐specific associations, and we were indeed able to identify and replicate multiple female‐specific associations in neurons and astrocytes. Additionally, our results provide broad support for previous suggestions of enhanced neuroprotective associations in neurons and enhanced risk associations in glial cells among females. Finally, our cell signaling analyses highlighted multiple cell–cell interactions that appeared to be altered in a sex‐specific manner in AD. Together, these findings provide strong evidence of sex‐specific transcriptomic drivers in the AD brain and provide a host of exciting sex‐specific targets for intervention that can be validated and prioritized in future mechanistic studies.

## AUTHOR CONTRIBUTIONS

Yiyang Wu and Timothy J. Hohman designed the study and wrote the manuscript. Yiyang Wu performed all the analyses. Kyle J. Travaglini, Mariano Gabitto, C. Dirk Keene, Philip L. De Jager, Vilas Menon, Julie A. Schneider, and David A. Bennett generated data, aided in the interpretation of results, and provided critical revision of the manuscript. Amy R. Dunn, Catherine C. Kaczorowski, and Logan Dumitrescu contributed to study design and provided critical revision of the manuscript. All authors approved the final manuscript.

## CONFLICT OF INTEREST STATEMENT

Timothy J. Hohman serves on the scientific advisory board of Vivid Genomics, serves as deputy editor for *Alzheimer's and Dementia: Translational Research and Clinical Intervention*, and as senior associate editor for *Alzheimer's and Dementia*. No other authors have competing interests. Author disclosures are available in the supporting information.

## CONSENT STATEMENT

All ROS/MAP participants signed an informed repository consent and an Anatomic Gift Act. Informed consent for SEA‐AD research brain donation was obtained according to protocols approved by the UW and Kaiser Permanente Washington Health Research Institute IRBs. Secondary analyses of the above datasets were approved by the Vanderbilt University Medical Center IRB.

## Supporting information



Supporting information

Supporting information

Supporting information

## Data Availability

Raw sequence and processed data of snRNA‐seq from ROS/MAP study are available at Synapse (https://www.synapse.org/#!Synapse:syn31512863). FASTQ files of SEA‐AD snRNA‐seq are available through controlled access at Sage Bionetworks (accession no. syn26223298). Processed SEA‐AD snRNA‐seq are available through the Open Data Registry in an AWS bucket (sea‐ad‐single‐cell‐profiling) as AnnData objects (h5ad files) and viewable on CELLxGENE and Allen Brain Cell Atlas (https://portal.brain‐map.org/atlases‐and‐data/bkp/abc‐atlas). All codes used for analysis discussed in the manuscript are available upon request.
