# Supplementary material for: Single‐cell landscape of sex‐specific drivers of Alzheimer's disease
Source: Alzheimers Dement. 2025 Dec 28;21(12):e71041. doi: 10.1002/alz.71041 (PMC12745176; doi:10.1002/alz.71041)
Supplement: Supplementary file 1 — Supporting information [file ALZ-21-e71041-s003.docx]

**Supplemental figures**

**Supplemental Fig 1: ROS/MAP Cell Composition influenced by sex or AD dementia diagnosis.**


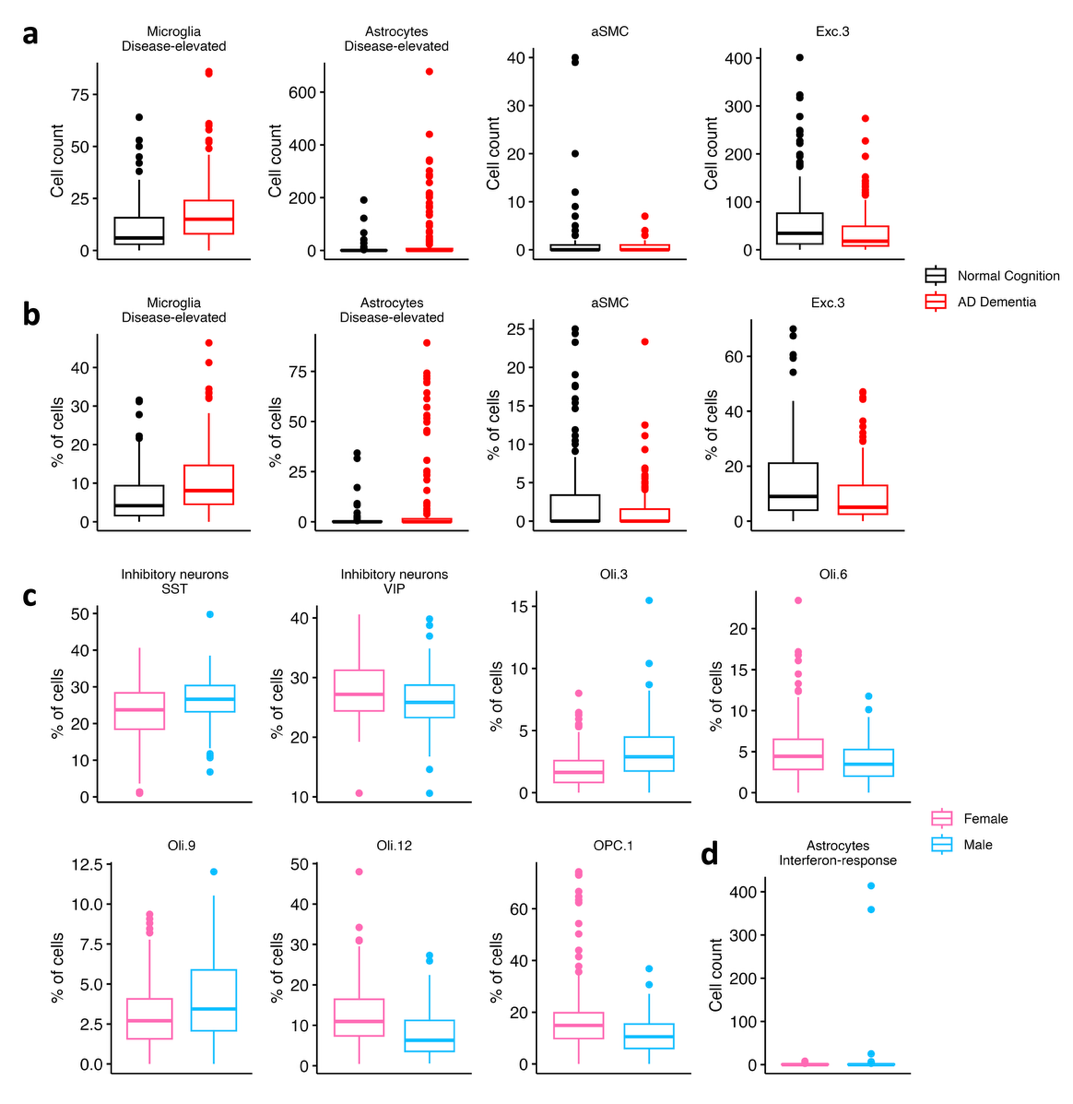


a, Boxplots showing the cell abundance of four cell subtypes that are influenced by AD dementia diagnosis. b, Boxplots showing cell fraction (percentage) of the four cell subtypes that are influenced by AD dementia diagnosis. c, Boxplots showing cell fraction (percentage) of the seven cell subtypes that are influenced by sex. d, Boxplots showing the cell abundance for interferon-response astrocytes that are influenced by sex.

**Supplemental Fig. 2: Six microglial-incoming signals comparison.**


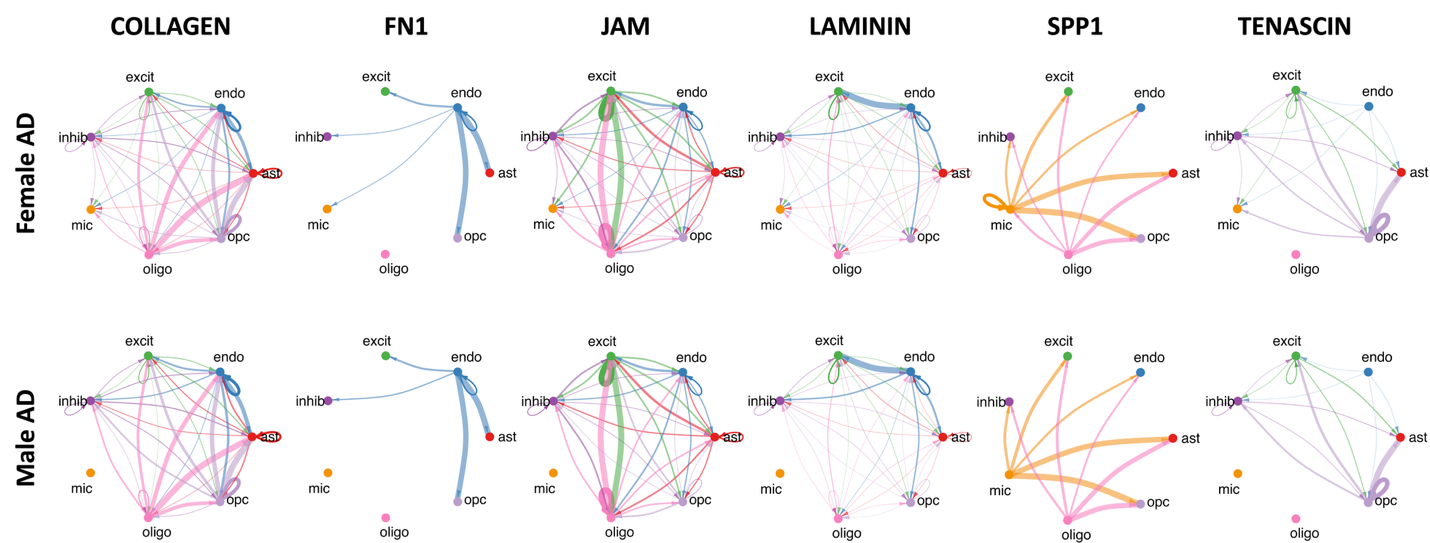


Chord diagram showing six signaling pathways in females and males with AD dementia. Males with AD dementia lacked meaningful incoming signals of each of these signals to microglia compared to females with AD dementia. Abbreviation: ast, astrocytes; excit, excitatory neurons; inhib, inhibitory neurons; mic, microglia; oligo, oligodendrocytes; opc, oligodendrocyte precursor cells; endo, endothelial cells. Each information flow represents combined interactions from all enriched ligand-receptor pairs.
